# Cervical Metastasis From Colorectal Cancer

**DOI:** 10.4021/wjon304w

**Published:** 2011-04-09

**Authors:** Elisabeth Chereau, Marcos Ballester, Julie Gonin, Benedicte Lesieur, Emile Darai

**Affiliations:** a Department of Gynecology-Obstetrics,Tenon Hospital, Assistance Publique des Hopitaux de Paris, CancerEst, Universite Pierre et Marie Curie Paris 6, France; b Department of Pathology, Tenon Hospital, Assistance Publique des Hopitaux de Paris, CancerEst, Universite Pierre et Marie Curie Paris 6, France

**Keywords:** Uterine cervix cancer, Metastasis, Immunohistochemical, Colorectal cancer

## Abstract

Metastatic carcinoma from colorectal cancer to the uterine cervix is rare. We report a case of metastatic carcinoma from a right colon cancer to the cervix with vaginal extension 3 years after primary treatment. Our report highlights the importance of immunohistochemical analysis to determine the origin of uterine cervix cancer in the event of adenocarcinoma in a patient with a history of colorectal cancer to adapt therapeutic strategy accordingly.

## Introduction

Common metastatic sites of colorectal cancer are the liver, lung, lymph nodes and peritoneum but metastasis to the uterine cervix is rare. We report here a case of metastatic carcinoma from a right colon cancer to the cervix with vaginal extension and a review of the literature on uterine cervix metastasis from colorectal cancer.

## Case Report

**Figure 1:**
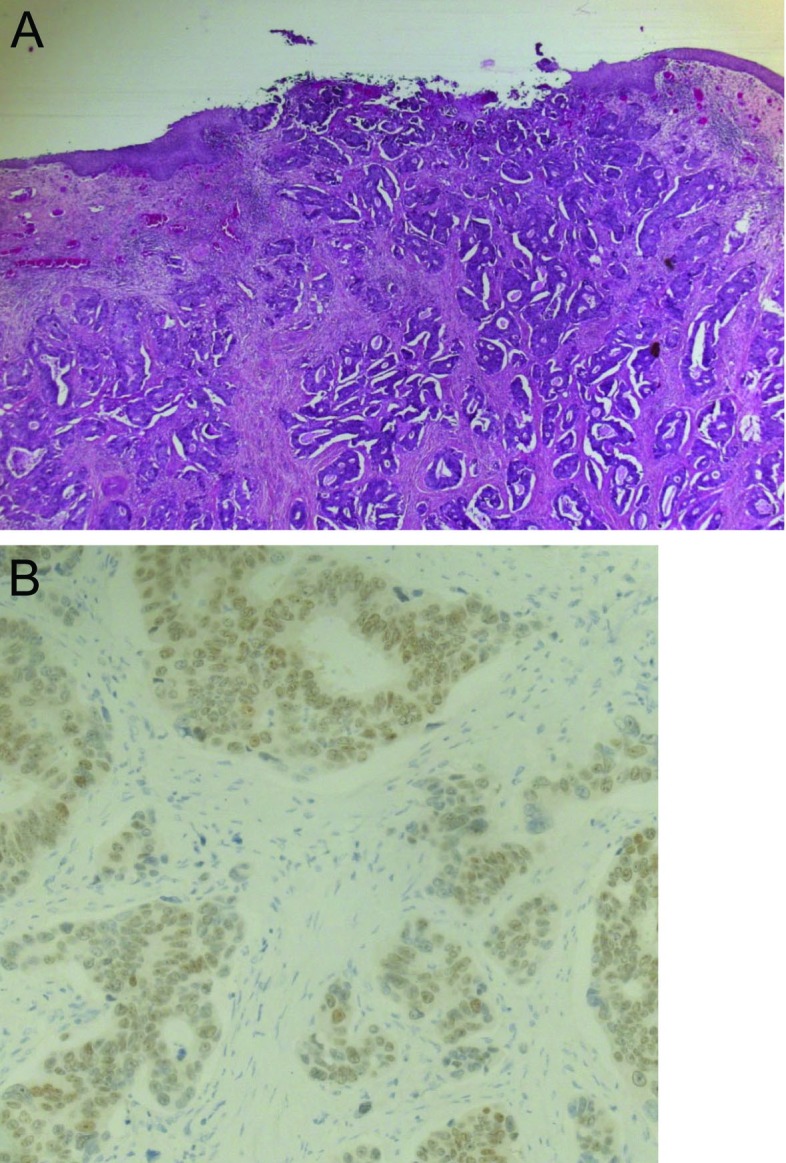
(A) Photo showing colonic metastatic lesion of the cervix from colon carcinoma in hematoxylin and eosin staining. (B) Photo showing immunohistochemical profile CDX2 positive in favor of colonic metastasis.

A 62-year-old woman was referred to our department for menopausal bleeding with suspicion of uterine cervix cancer. Her medical history revealed that she had undergone laparotomic surgery for a right colon cancer with lymph node metastasis 3 years previously. She received adjuvant chemotherapy (folfox and bevacizumab) at that time. Postoperative physical examination and CT scan did not detect relapse of colon carcinoma and serum CEA was normal. There were no other salient features to her medical history. On physical examination in our department, a cervical lesion with vaginal involvement measuring 30 mm was found. Rectal digital examination found no parametrial or rectal involvement. Cervical biopsy histology showed a poorly differentiated adenocarcinoma. MRI confirmed the presence of a cervical lesion with extension to the vagina but independent from the rectum confirming a FIGO stage IIA1. According to our protocol, the patient underwent a peritoneal washing and a radical hysterectomy with bilateral salpingo-oophorectomy and bilateral pelvic lymphadenectomy by laparotomy. After surgery, the patient had no residual disease. The postoperative course was normal. Histology confirmed a poorly differentiated adenocarcinoma of the uterine measuring 15 mm and limited to the uterine cervix without vaginal extension. Immunohistochemical (IHC) profile (cytokeratin 20 and CDX2 positive but cytokeratin 7 negative) revealed colic cancer metastasis (Fig. 1). The peritoneal washing was negative. No metastatic involvement was found among the 15 nodes removed or in the fallopian tubes and ovaries but there was proximal parametrial involvement. Given these histological findings an additional colonoscopy was performed to exclude recurrence of colorectal cancer. Chest and abdomino-pelvic CT scan found no evidence of metastasis and this was confirmed by a negative Pet-FDG. The Oncology Committee recommended adjuvant chemotherapy adapted for colorectal cancer and pelvic radiotherapy.

## Discussion

The uterus, and especially the uterine cervix, is a rare location of colorectal metastasis: involvement of the uterus represents less than 10% of all cases of metastases to the female genital tract from extragenital cancers (3.4% for the uterine cervix alone). Extragenital tumors most often metastasizing to the uterine cervix or corpus generate from the breast (42.2%), stomach (18.5%), pancreas (5.2%), lung (4.6%), urinary bladder and kidney (4.6%), gallbladder (2.3%) and cutaneous melanoma (1.7%) [1]. Involvement of the uterine cervix is usually by direct extension of extragenital neoplasia and this is relatively frequent. However, the reason why metastatic carcinoma to the cervix alone through hematogenous or lymphatic spread is so rare is because of its fibrous tissue content, its small size and its relatively limited blood flow. Moreover, the lymphatic vessels of the pelvis all drain away from the cervix [2].

In the literature, only a few articles have reported uterine cervix metastasis. Nakagami et al reported a case of uterus cervix metastasis from rectal cancer and reviewed 27 cases of cervical metastases from colorectal carcinomas which constitutes the largest review to date [3]. The interval between primary carcinoma and the uterine metastatic disease diagnosis was 17 months with a range from 0 to 60 months. Gynecological symptoms were vaginal bleeding, pelvic pain, mass and vaginal discharge. The survival period after the diagnosis of the secondary deposit was 11 months, ranging from 1 to 60 months [4]. Nevertheless in most of the cases, cervix metastasis occurred after rectal carcinoma whereas in our case it occurred after right colon cancer which is unusual.

The present case report addresses several issues. First, the pathological mechanism was involved in the occurrence of a uterine cervix metastasis. The absence of metastasis at CT scan and Pet-FDG suggests that the uterine cervix was involved via metastasis in the peritoneal pouch of Douglas through the torus uterinum or posterior vaginal cuff subsequent to cell dissemination during the first laparotomy. Second, in patients with prior colon carcinoma, the practitioner should be aware of a possible metastasis to the uterine cervix on detection of an adenocarcinoma. In this specific setting, the contribution of IHC is crucial to differentiate a genital from extragenital origin of the uterine cervix tumor. Finally, preoperative diagnosis of the extragenital origin of the uterine cervix metastasis could lead to a different therapeutic strategy including an initial chemotherapy with drugs specific to colorectal carcinoma and then pelvic radiotherapy. The risks of a radical hysterectomy would thus be minimized.

In conclusion, in the event of adenocarcinoma in the uterine cervix in a patient with a history of colorectal cancer, the possibility of an extragenital origin should be discussed. The therapeutic strategy should be adapted accordingly if the extragenital origin of the tumor is confirmed by IHC.
